# New 7-[4-(4-(un)Substituted)piperazine-1-carbonyl]-piperazin-1-yl] Derivatives of Fluoroquinolone: Synthesis and Antimicrobial Evaluation

**DOI:** 10.3390/molecules18077557

**Published:** 2013-06-27

**Authors:** Po-Ting Chen, Wen-Po Lin, An-Rong Lee, Ming-Kuan Hu

**Affiliations:** 1 School of Pharmacy, National Defense Medical Center, 161 Min-Chuan East Road, Section 6, Taipei 114, Taiwan; E-Mails: wawaspider@yahoo.com.tw (P.-T.C.); lar@ndmctsgh.edu.tw (A.-R.L.); 2 Department of Microbiology and Immunology, National Defense Medical Center, 161 Min-Chuan East Road, Section 6, Taipei 114, Taiwan; E-Mail: ae3843@ndmctsgh.edu.tw

**Keywords:** antimicrobial agents, ciprofloxacin, ciprofloxacin-resistant *Pseudomonas aeruginosa*, methicillin-resistant *Staphylococcus aureus*

## Abstract

Fluoroquinolones have been a class of important synthetic antimicrobial agents broadly and effectively used in clinic for infectious diseases. In this study, the synthesis of a range of fluoroquinolone derivatives with 4-(carbopiperazin-1-yl)piperazinyl moieties at the C7 position and their inhibition of bacterial pathogens commonly disseminated in hospital environment were described. The results indicated that a 7-[4-(4-(benzoyl)carbopiperazin-1-yl)]piperazinyl derivative **5h** and two 7-[4-(4-(benzenesulfonyl)carbopiperazin-1-yl)]piperazinyl derivatives **5k** and **5l** showed more promising growth inhibition of ciprofloxacin-resistant *P. aeruginosa* (CRPA) with MIC values as low as 16 μg/mL which is 16-fold more potent than ciprofloxacin, while most of other derivatives maintained potency against methicillin-resistant *Staphylococcus aureus* (MRSA).

## 1. Introduction

Despite advances in drug development among pharmaceutical companies, it has proved relatively difficult to achieve breakthroughs in the discovery of new antimicrobial agents with new targets. Currently, one of the practical approaches to these challenges is direct manipulation of the structure of exsiting antibacterial agents to improve antimicrobial potency and efficacy. Fluoroquinolones are a type of important synthetic antibacterial agents used broadly and effectively in clinic for infectious diseases [1,2]. They possess excellent activities against Gram-negative and relatively moderate against Gram-positive bacteria. Although certain adverse events still remain during the use of fluoroquinolones for therapies, fluoroquinolones are still one of the major antimicrobial agents with many advances for clinical use, and much continuous effort has gone into the structural modification of the fluoroquinolone framework to provide newer congeners with improved potency and efficacy to conquer the fluoroquinolone-resistant pathogens commonly encountered in the hospital environment [3,4,5].

Based on the struture-activity relationships of fluoroquinolones that have been well addressed, substituents at the 7-position of the fluoroquinolone skeleton greatly influence their antimicrobial spectrum, potency, and even safety [6]. With the piperazinyl moieties in ciprofloxacin, levofloxacin, and sparfloxacin, the basicity and lipophilicity of each moiety dominate their ability to penetrate into cell walls and widen their activity spectrum. From the structure-activity point of view, the variety of piperazinyl substituents at the 7-position of fluoroquinolone agents has disclosed the looseness of the binding pocket of the targeted type II topoisomerases and established the groundwork for further modification toward new fluoroquinolone agents useful against certain clinically resistant organisms [7,8,9,10].

In our research program on antimicrobial agents, we recently developed a series of ciprofloxacin derivatives bearing a 3-carboxamate moiety, which were found to exhibit comparable activities against certain Gram-negative organisms [11]. In this study, we manipulated the piperazinyl moiety at the 7-position of ciprofloxacin with an additional carbopiperazinyl group and introduced various *N*-benzoyl and *N*-benzenesulfonyl substituents to the carbopiperazinyl moiety to improve the relative antimicrobial activities against certain resistant species commonly disseminated in the hospital environment.

## 2. Results and Discussion

### 2.1. Synthesis

**Scheme 1:**
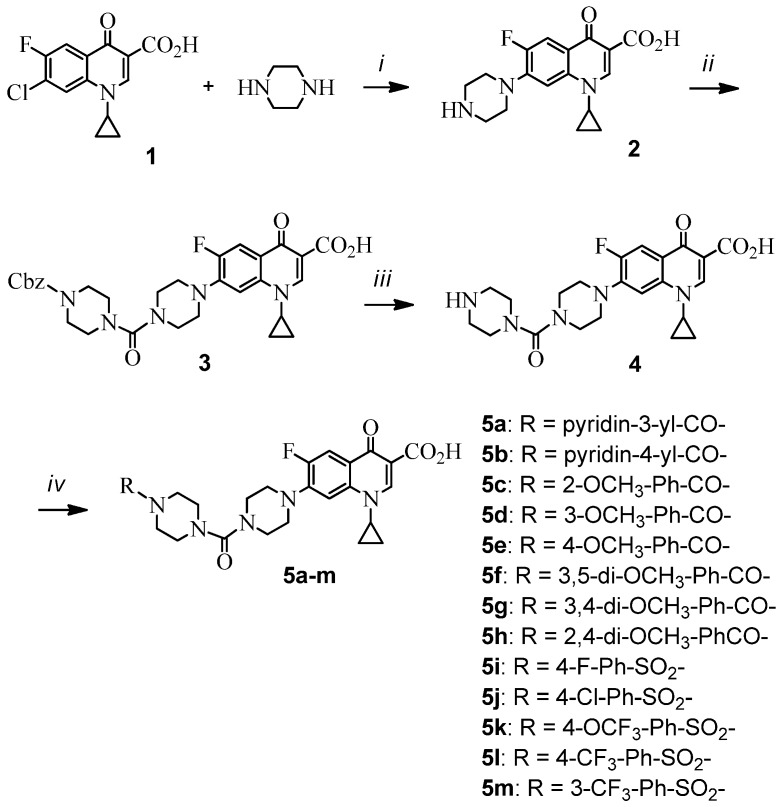
Synthesis of 7-(4-substituted 4-(carbopiperazin-1-yl))piperazinyl derivatives of fluoroquinolone.

The synthesis of the novel 7-(4-carbopiperazin-1-yl)piperazinyl derivatives of fluoroquinolone was described in Scheme 1. Ciprofloxacin (**2**) was economically prepared from condensation of 1-cyclopropyl-7-chloro-6-fluoro-1,4-dihydro-4-oxoquinoline-3-carboxylic acid (**1**) and piperazine in dimethylsulfoxide with the assistance of microwave irradiation (100 W, 2 h) [12]. The manipulation of the piperazinyl moiety at the 7-position of ciprofloxacin with an additional carbopiperazinyl group was conducted *in situ* with triphosgene and *N*-protected piperazine to give 7-(4-carbopiperazin-1-yl) intermediate **3** in moderate yields. Heterogenous hydrogenation (5% Pd on activated carbon) of **3** followed by aroylation or benzenesulfonation of the resulting free amine led to a range of 7-[4-(4-(aroyl)carbopiperazin-1-yl)]piperazinyl and 7-[4-(4-(benzenesulfonyl)carbopiperazin-1-yl)]- piperazinyl derivatives **5a–m** in good yields (55~85%) after purification by silica gel chromatography. The chemical structures and log *P* values of these new ciprofloxacin derivatives are listed in Table 1.

*Reagents and conditions*: (*i*) microwaves (100 W, 2h), dimethylsulfoxide (*ii*) triphosgene, triethylamine, *N*-benzyloxycarbonyl-piperazine (*iii*) H_2(g)_, 5% Pd/C; (*iv*) aroyl- or benzenesulfonyl chlorides, triethylamine, room temperature.

### 2.2. Inhibition of Bacterial Growth

For the evaluation of the antibacterial activities of these 7-(4-carbopiperazin-1-yl) derivatives of ciprofloxacin, the minimal inhibitory concentrations (MICs) that prevent visible growth of bacteria were determined by a standard broth microdilution method [13]. The MIC values of the synthetic ciprofloxacin derivatives along with the standard drugs tested according to this approach are reported in Table 2. In the first phase of screening against conventional Gram-negative bacteria, most of the 7-(4-carbopiperazin-1-yl) derivatives exhibited substantial activities, similar to the prevailing fluoroquinolones, against *E. coli*, *beta*-lactamase-producing *E. coli* (*pUC18*), and *P. aeruginosa*, with MICs of less than 0.016 μg/mL. We distinctively observed the ability of these new derivatives against a clinically isolated ciprofloxacin-resistant (*cipro^r^*) strain of *P. aeruginosa* (CRPA) and found that five ciprofloxacin derivatives (compounds **5h–l**) exhibited improved growth inhibition against CRPA compared to ciprofloxacin, with MICs in the 16 μg/mL to 64 μg/mL range. When tested on the Gram-positive *B. subtilis*, two benzoyl (compounds **5f**, **5h**) and three benzenesulfonyl derivatives (compounds **5i–k**) displayed moderate *in vitro* activities, with MICs ranging from 1 μg/mL to 4 μg/mL, while pyridocarbonyl analogues **5a** and **5b** and methoxybenzoyl analogues **5d** and **5e** were apparently inactive (MIC ≥ 256 μg/mL). However, most of the synthesized derivatives showed excellent activities similar to ciprofloxacin against MRSA (MIC < 0.016 μg/mL). Only four of them (compounds **3**, **4**, and **5l–m**) displayed moderate potency (MIC values, 4 μg/mL~16 μg/mL). These results revealed that the increased lipophilicity with the additional 4-((4-substituted)carbopiperazin-1-yl) moieties at the 7-position of ciprofloxacin improves the abilities of the fluoroquinolone derivatives to penetrate the microbial cell wall, thus improving their antimicrobial activities, especially against CRPA and also maintaining potency on the tested Gram-negative species. Recently, Huang and co-workers reported that 7-triazolylpiperidinyl fluoroquinolone derivatives showed comparable antibacterial activity compared to ciprofloxacin [14]. Our series of ciprofloxacin derivatives featuring an additional carbopiperazinyl moiety at the 7-position exhibited potent antimicrobial activities against certain clinical isolates of Gram-negative strains.

**Table 1:** Structure, yield, and lipophilicity of the synthesized ciprofloxacin derivatives. 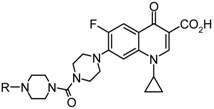

Compound	R	yield	lo*g P* ^a^	Compound	R	yield	lo*g P* ^a^
**3**	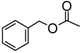	55%	2.42	**5g**	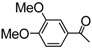	78%	1.99
**4**	ᴴ	80%	0.08	**5h**	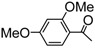	65%	2.10
**5a**	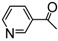	62%	1.19	**5i**	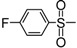	76%	1.83
**5b**	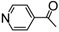	55%	0.99	**5j**	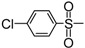	78%	2.33
**5c**	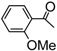	67%	1.97	**5k**	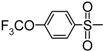	80%	2.84
**5d**	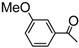	82%	1.98	**5l**	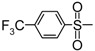	78%	2.61
**5e**	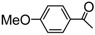	85%	2.17	**5m**	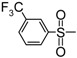	76%	2.81
**5f**	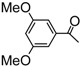	73%	1.90	**Ciprofloxacin**		75%	0.50

^a^ Calculated log *P* values were obtained from the ALogPS 2.1 program [15].

**Table 2:** *In vitro* MIC values of ciprofloxacin derivatives against certain G(+)- and G(−)-strains.

Compound	MIC (μg/mL)					
	*E. coli*	*E. coli (pUC18)*	*P. aeru.*	*P. aeru. (cipro^r^)*	*MRSA*	*B. subtilis*
**3**	0.063	<0.016	<0.016	>256	64	256
**4**	0.063	<0.016	<0.016	>256	64	>256
**5a**	<0.016	<0.016	<0.016	>256	<0.016	>256
**5b**	<0.016	<0.016	<0.016	>256	<0.016	>256
**5c**	<0.016	<0.016	<0.016	128	<0.016	16
**5d**	<0.016	<0.016	<0.016	>256	<0.016	>256
**5e**	<0.016	<0.016	<0.016	>256	<0.016	>256
**5f**	<0.016	<0.016	<0.016	128	<0.016	4
**5g**	<0.016	<0.016	<0.016	>256	<0.016	>256
**5h**	<0.016	<0.016	<0.016	16	<0.016	4
**5i**	0.063	0.063	<0.016	64	<0.016	4
**5j**	<0.016	<0.016	<0.016	64	<0.016	1
**5k**	<0.016	<0.016	<0.016	16	<0.016	1
**5l**	<0.016	<0.016	<0.016	16	4	>256
**5m**	<0.016	<0.016	<0.016	>256	64	16
**Ciprofloxacin**	<0.016	<0.016	<0.016	>256	<0.016	<0.016
**Norfloxacin**	<0.016	<0.016	<0.016	>256	<0.016	0.5

In order to examine whether these derivatives possessed bactericidal activities against these selected bacterial species, we further measured the minimum bactericidal concentrations (MBCs) based on each MIC by a dilution method as previously described [16]. The MBC values of these derivatives according to the method are given in Table 3. Most of the synthesized compounds generally showed less bactericidal activities against Gram-negative bacteria than the prevailing ciprofloxacin and norfloxacin, yet two benzenesulfonyl derivatives (compound **5j** and **5k**) exhibited substantial potency, with MBCs less than 0.016 μg/mL against *E. coli*. From the screening on the clinically emerging β-lactamase-producing *E. coli*, the MBCs of the derivatives **5a** and **5c–f** were in the range from 0.016 μg/mL to 0.063 μg/mL. These results indicated that the synthesized derivatives with 4-benzoyl substituents at the 7-(4-carbopiperazin-1-yl) position showed stronger bactericidal effects against Gram-negative species than those analogs with benzenesulfonyl moieties. Concerning the screening on Gram-positive bacterial strains, most derivatives showed only moderate bactericidal activities on *B. subtilis*, probably because the unique capsular membrane component of *B. subtilis* was an obstacle to the membrane penetration of the tested compounds and resulted in the observed decreased bactericidal activities. They were also less active than ciprofloxacin against MRSA. The results suggested that in these derivatives the presence of additional moieties at the 7-position of ciprofloxacin seems not to be beneficial to their bactericidal abilities against the tested Gram-positive species.

**Table 3:** *In vitro* MBC values of ciprofloxacin derivatives against certain G(+)- and G(-)-strains.

	MBC (μg/mL)					
	*E. coli*	*E. coli (pUC18)*	*P. aeru.*	*P. aeru. (cipro^r^)*	*MRSA*	*B. subtilis*
**3**	128	0.25	256	>256	>256	64
**4**	128	0.25	256	>256	128	>256
**5a**	4	<0.016	>256	>256	>256	16
**5b**	0.25	1	>256	>256	>256	16
**5c**	1	<0.016	>256	256	>256	16
**5d**	16	0.063	>256	>256	>256	128
**5e**	4	0.063	>256	>256	128	64
**5f**	16	0.063	>256	256	>256	16
**5g**	16	0.25	>256	>256	>256	>256
**5h**	16	0.25	>256	>256	>256	16
**5i**	16	0.25	>256	>256	128	4
**5j**	<0.016	1	>256	>256	>256	4
**5k**	<0.016	1	>256	>256	>256	4
**5l**	64	1	>256	>256	>256	>256
**5m**	128	0.063	>256	>256	>256	16
**Ciprofloxacin**	<0.016	<0.016	0.125	>256	0.031	0.125
**Norfloxacin**	<0.016	<0.016	4	>256	4	0.5

## 3. Experimental

### 3.1. General

All reagents and solvents were commercial materials and were used directly unless otherwise noted. DMF was dehydrated over 4 Å molecular sieves. Reactions were monitored by thin layer chromatography using Echo silica gel F254 plates visualized under UV irradiation along with staining with phosphomolybdic acid/heat, or iodine. Melting points were recorded on a Thomas Hoover capillary melting point apparatus in open capillary tubes and are uncorrected. NMR spectra were recorded on a Varian Gemini instrument at 300 MHz for ^1^H and at 75 MHz for ^13^C. Fast atom bombardment mass spectra (FABMS) were acquired on a Finnigan Mat 95S mass spectrometer. Chromatography refers to flash chromatography on silica gel (silica gel 60, 230–400 mesh ASTM, E. Merck, Darmstadt, Germany).

### 3.2. Synthesis

*7-(4-[4-(Benzyloxycarbonyl)piperazino]carbopiperazino)-1-cyclopropyl-6-fluoro-4-oxo-1,4-dihydro-3-quinolinecarboxylic acid* (**3**). A solution of *N*-(benzyloxycarbonyl)piperazine (220 mg, 1 mmol) in dichloromethane (10 mL) was slowly added to the stirred solution of triphosgene (110 g, 0.37 mmol) in dichloromethane (2 mL) over a period of 30 min using a syringe pump. After 30 further min of stirring, a solution of **2** (398 mg, 1.2 mmol) and diisopropylethylamine (DIEA, 0.38 mL, 2.2 mmol) in DCM/EtOH (120 mL, 4:1) was added in one portion. The reaction mixture was stirred for 2 hours at room temperature. After evaporation of solvent under vacuum, the residue was purified by silica gel chromatography to give **3** (318 mg, 55%); tlc R_f_ = 0.30 (DCM/EtOH = 20:1); mp 215–216 °C; UV λ_max_ (DCM/EtOH = 3:2) nm (log ε) 240.4 (9.22); ^1^H-NMR (DMSO-*d_6_*) δ 8.67 (s, 1H, C_2_-H), 7.90 (d, *J* = 13.2 Hz, 1H, C_5_-H), 7.56 (d, *J* = 7.5 Hz, 1H, C_8_-H), 7.37–7.31 (m, 5H, Ar-H), 5.09 (s, 2H, -O-CH_2_-Ar), 3.78–3.82 (m, 1H, cyclopropyl), 3.15–3.40 (m, 16H, piperazinyl), 1.17–1.32 (m, 4H, cyclopropyl); ^13^C-NMR (CDCl_3_) δ 8.1, 35.2, 41.4, 41.5, 44.3, 44.6, 48.1, 49.3, 49.6, 50.3, 69.5, 105.4, 108.4, 111.1, 112.8, 118.8, 127.3, 128.2, 128.8, 129.6, 136.9, 137.8, 146.5, 149.8, 153.4, 161.2, 165.3, 168.6, 176,2; FABMS: *m/z* 578 [M+H]^+^.

*1-Cyclopropyl-6-fluoro-4-oxo-7-[4-(piperazin-1-yl)carbopiperazino]-1,4-dihydro-3-quinolinecarboxylic acid* (**4**). To a solution of **3** (289 mg, 0.5 mmol) and 10% Pd/C in DCM/EtOH (50 mL, 1:1) was charged with H_2_ at 1 atm and stirred at room temperature for 1 h. The catalyst was filtered off through celite. After evaporation of solvent under vacuum, the residue was recrystallized from DCM/acetone to give **4** (177 mg, 80%); tlc R_f_ = 0.10 (DCM/EtOH = 9:1); mp 196–197 °C; UV λ_max_ (DCM/EtOH = 3:2) nm (log ε) 238.8 (9.11); ^1^H-NMR (DMSO-*d_6_*) δ 9.10 (s, 1H, br, CH_2_-NH-CH_2_), 8.77 (s, 1H, C_2_-H), 7.91 (d, *J* = 13.2 Hz, 1H, C_5_-H), 7.55 (d, *J* = 7.5 Hz, 1H, C_8_-H), 3.78–3.82 (m, 1H, cyclopropyl), 3.08–3.40 (m, 16H, piperazinyl), 1.16–1.31 (m, 4H, cyclopropyl); ^13^C-NMR (CDCl_3_) δ 8.1, 35.2, 40.2, 41.6, 43.6, 44.6, 48.2, 49.2, 49.6, 50.3, 106.2, 108.4, 112.8, 118.8, 137.8, 146.5, 149.8, 153.4, 161.2, 166.6, 176,2; FABMS: m/z 444 [M+H]^+^.

*General procedure for the synthesis of 1-cyclopropyl-6-fluoro-4-oxo-7-(4-[4-(substituted benzoyl or benzenesulfonyl) piperazino]carbopiperazino)-1,4-dihydro-3-quinolinecarboxylic acids* **5a**–**m**.

To a solution of amine **4** (0.67 g, 1.5 mmol) in DCM/EtOH (150 mL, 4:1) was added triethylamine (0.4 mL, 3.0 mmol) and the appropriate aroyl or benzenesulfonyl halide (1.2 mmol). The mixture was stirred at room temperature under argon for several hours depending on the completion of the reaction, which was checked by tlc. After evaporation of solvent under vacuum, the residue was purified by silica gel chromatography and recrystallized from an appropriate solvent to give the title products.

*1-Cyclopropyl-6-fluoro-4-oxo-7-(4-[4-(3-pyridylcarbonyl)piperazino]carbopiperazino)-1,4-dihydro-3-quinolinecarboxylic acid* (**5a**). Amine **4** (0.67 g, 1.5 mmol) was treated with nicotinyl chloride (0.17 g, 1.2 mmol) to give **5a** (0.41 g, 62%) as a white solid; tlc R_f_ = 0.18 (DCM/EtOH = 20 : 1); mp 219–220 °C; UV λ_max_ (DCM/EtOH = 3:2) nm (log ε): 242.6 (9.20); ^1^H-NMR (CDCl_3_) δ 8.63–8.65 (m, 2H, Ar-H), 8.61 (s, 1H, C_2_-H), 7.84 (d, *J* = 12.9 Hz, 1H, C_5_-H), 7.73–7.76 (m, 1H, Ar-H), 7.35–7.39 (m, 1H, Ar-H), 7.32 (d, *J* = 7.2 Hz, 1H, C_8_-H), 3.76–3.80 (m, 1H, cyclopropyl), 3.32–3.53 (m, 16H, piperazinyl), 1.17–1.40 (m, 4H, cyclopropyl); ^13^C-NMR (CDCl_3_) δ 8.1, 35.3, 41.3, 41.9, 44.4, 44.8, 48.8, 49.2, 49.6, 50.1, 105.1, 107.2, 109.4, 112.0, 112.3, 119.8, 127.1, 138.0, 144.6, 145.0, 147.2, 149.8, 153.6, 160.6, 166.0, 168.8, 176,6; FABMS: m/z 549 [M+H]^+^; HRFABMS: calcd for C_28_H_30_FN_6_O_5_ [M+H]^+^ 549.2264, found 549.2268. 

*1-Cyclopropyl-6-fluoro-4-oxo-7-(4-[4-(4-pyridylcarbonyl)piperazino]carbopiperazino)-1,4-dihydro-3-quinolinecarboxylic acid* (**5b**). Amine **4** (0.67 g, 1.5 mmol) was treated with isonicotinyl chloride (0.17 g, 1.2 mmol) to give **5b** (0.36 g, 55%) as a white solid; tlc R_f_ = 0.18 (DCM/EtOH = 20:1); mp 217–218 °C; UV λ_max_ (DCM/EtOH = 3:2) nm (log ε): 239.4 (9.20); ^1^H-NMR (CDCl_3_) δ 8.74–8.65 (m, 2H, Ar-H), 8.67 (s, 1H, C_2_-H), 7.91 (d, J = 12.9 Hz, 1H, C_5_-H), 7.34 (d, J = 7.2 Hz, 1H, C_8_-H), 7.30–7.35 (m, 2H, Ar-H), 3.78–3.82 (m, 1H, cyclopropyl), 3.32–3.54 (m, 16H, piperazinyl), 1.18–1.40 (m, 4H, cyclopropyl); ^13^C-NMR (CDCl_3_) δ 8.2, 35.3, 41.6, 41.9, 44.3, 44.5, 47.9, 49.3, 49.6, 50.4, 105.8, 106.9, 108.7, 111.3, 112.2, 118.8, 127.3, 137.8, 144.2, 146.5, 147.4, 149.8, 153.4, 161.2, 165.3, 168.2, 176,3; FABMS: m/z 549 [M+H]^+^; HRFABMS: calcd for C_28_H_30_FN_6_O_5_ [M+H]^+^ 549.2264, found 549.2262. 

*1-Cyclopropyl-6-fluoro-7-(4-[4-(2-methoxybenzoyl)piperazino]carbopiperazino)-4-oxo-1,4-dihydro-3-quinolinecarboxylic acid* (**5c**). Amine **4** (0.67 g, 1.5 mmol) was treated with 2-methoxybenzoyl chloride (0.2 g, 1.2 mmol) to give **5c** (0.46 g, 67%) as a white solid; tlc R_f_ = 0.33 (DCM/EtOH = 20:1); mp 240–241 °C; UV λ_max_ (DCM/EtOH = 3:2) nm (log ε): 241.8 (9.27); ^1^H-NMR (CDCl_3_) δ 8.66 (s, 1H, C_2_-H), 7.90 (d, J = 12.9 Hz, 1H, C_5_-H), 7.34 (d, J = 7.2 Hz, 1H, C_8_-H), 6.89–7.38 (m, 4H, Ar-H), 3.82 (s, 3H, -OCH_3_), 3.78–3.85 (m, 1H, cyclopropyl), 3.26–3.53 (m, 16H, piperazinyl), 1.17–1.39 (m, 4H, cyclopropyl); ^13^C NMR (CDCl_3_) δ 8.2, 35.3, 41.6, 41.9, 44.3, 44.5, 47.9, 49.3, 49.6, 50.4, 54.2, 107.1, 108.7, 111.3, 112.2, 114.6, 118.8, 120.8, 127.3, 128.2, 129.2, 137.8, 146.5, 149.8, 153.4, 160.2, 161.2, 165.3, 168.6, 176,2; FABMS: m/z 578 [M+H]^+^; HRFABMS: calcd for C_30_H_33_FN_5_O_6_ [M+H]^+^ 578.2417, found 578.2420. 

*1-Cyclopropyl-6-fluoro-7-(4-[4-(3-methoxybenzoyl)piperazino]carbopiperazino)-4-oxo-1,4-dihydro- 3-quinolinecarboxylic acid* (**5d**). Amine **4** (0.67 g, 1.5 mmol) was treated with 3-methoxybenzoyl chloride (0.2 g, 1.2 mmol) to give **5d** (0.57 g, 82%) as a white solid; tlc R_f_ = 0.34 (DCM/EtOH = 20:1); mp 236–237 °C; UV λ_max_ (DCM/EtOH = 3:2) nm (log ε): 240.7 (9.27); ^1^H-NMR (CDCl_3_) δ 8.70 (s, 1H, C_2_-H), 7.95 (d, J = 12.9 Hz, 1H, C_5_-H), 7.35 (d, J = 7.2 Hz, 1H, C_8_-H), 7.29–7.35 (m, 1H, Ar-H), 6.92–6.98 (m, 3H, Ar-H), 3.82 (s, 3H, -OCH_3_), 3.78–3.82 (m, 1H, cyclopropyl), 3.26–3.54 (m, 16H, piperazinyl), 1.1–1.40 (m, 4H, cyclopropyl); ^13^C-NMR (CDCl_3_) δ 8.2, 35.1, 41.4, 41.9, 44.3, 44.5, 47.9, 49.2, 49.6, 50.3, 53.7, 107.2, 107.9, 111.5, 112.2, 114.6, 118.8, 127.3, 128.2, 129.2, 137.8, 146.4, 149.8, 153.4, 160.2, 161.2, 165.6, 168.4, 176,2; FABMS: m/z 578 [M+H]^+^, HRFABMS: calcd for C_30_H_33_FN_5_O_6_ [M+H]^+^ 578.2417, found 578.2413. 

*1-Cyclopropyl-6-fluoro-7-(4-[4-(4-methoxybenzoyl)piperazino]carbopiperazino)-4-oxo-1,4-dihydro-3-quinolinecarboxylic acid* (**5e**). Amine **4** (0.67 g, 1.5 mmol) was treated with 4-methoxybenzoyl chloride (0.2 g, 1.2 mmol) to give **5e** (0.59 g, 85%) as a white solid; tlc R_f_ = 0.34 (DCM/EtOH = 20:1); mp 224–225 °C; UV λ_max_ (DCM/EtOH = 3:2) nm (log ε): 239.0 (9.23); ^1^H-NMR (CDCl_3_) δ 8.60 (s, 1H, C_2_-H), 7.83 (d, *J* = 12.9 Hz, 1H, C_5_-H), 7.35 (dd, *J* = 6.9, 2.1 Hz, 2H, Ar-H), 7.32 (d, *J* = 7.2 Hz, 1H, C_8_-H), 6.88 (dd, *J* = 6.9, 2.1 Hz, 2H, Ar-H), 3.80 (s, 3H, -OCH_3_), 3.78–3.82 (m, 1H, cyclopropyl), 3.30–3.62 (m, 16H, piperazinyl), 1.16–1.40 (m, 4H, cyclopropyl); ^13^C-NMR (CDCl_3_) δ 8.2, 35.2, 41.7, 41.2, 44.6, 44.9, 47.3, 49.2, 49.6, 50.3, 53.2, 107.3, 107.9, 110.8, 111.6, 114.7, 118.8, 127.5, 128.2, 129.2, 137.8, 146.4, 148.2, 153.4, 160.2, 161.6, 165.8, 168.3, 176,2; FABMS: m/z 578 [M+H]^+^, HRFABMS: calcd for C_30_H_33_FN_5_O_6_ [M+H]^+^ 578.2417, found 578.2422.

*1-Cyclopropyl-7-(4-[4-(3,5-dimethoxybenzoyl)piperazino]carbopiperazino)-6-fluoro-4-oxo-1,4-dihydro-3-quinolinecarboxylic acid* (**5f**). Amine **4** (0.67 g, 1.5 mmol) was treated with 3,5-dimethoxybenzoyl chloride (0.24 g, 1.2 mmol) to give **5f** (0.53 g, 73%) as a white solid; tlc R_f_ = 0.38 (DCM/EtOH = 20:1); mp 233–234 °C; UV λ_max_ (DCM/EtOH = 3:2) nm (log ε): 240.4 (9.25); ^1^H-NMR (CDCl_3_) δ 8.61 (s, 1H, C_2_-H), 7.84 (d, *J* = 12.9 Hz, 1H, C_5_-H), 7.32 (d, *J* = 6.9 Hz, 1H, C_8_-H), 6.46–6.48 (m, 3H, Ar-H), 3.77 (s, 6H, -OCH_3_), 3.78–3.82 (m, 1H, cyclopropyl), 3.30–3.65 (m, 16H, piperazinyl), 1.17–1.39 (m, 4H, cyclopropyl); ^13^C-NMR (CDCl_3_) δ 8.1, 35.2, 41.6, 41.2, 44.6, 44.8, 47.3, 49.5, 49.2, 50.3, 53.4, 53.8, 106.8, 107.9, 110.8, 112.4, 114.7, 118.9, 127.5, 128.1, 137.8, 146.4, 148.2, 153.4, 159.8, 160.4, 161.4, 165.8, 168.2, 176,2; FABMS: m/z 608 [M+H]^+^, HRFABMS: calcd for C_31_H_35_FN_5_O_7_ [M+H]^+^ 608.2523, found 608.2527.

*1-Cyclopropyl-7-(4-[4-(3,4-dimethoxybenzoyl)piperazino]carbopiperazino)-6-fluoro-4-oxo-1,4-dihydro-3-quinolinecarboxylic acid* (**5g**). Amine **4** (0.67 g, 1.5 mmol) was treated with 3,4-dimethoxybenzoyl chloride (0.24 g, 1.2 mmol) to give **5g** (0.57 g, 78%) as a white solid; tlc R_f_ = 0.38 (DCM/EtOH = 20:1); mp 257–258 °C; UV λ_max_ (DCM/EtOH = 3:2) nm (log ε): 243.8 (9.28); ^1^H-NMR (CDCl_3_) δ 8.72 (s, 1H, C_2_-H), δ 7.98 (d, J = 12.9 Hz, 1H, C_5_-H), δ 7.36 (d, J = 7.2 Hz, 1H, C_8_-H), δ 6.99 (d, J = 1.8 Hz, 1H, Ar-H), δ 6.98 (dd, J = 9.9 Hz, 1.8 Hz, 1H, Ar-H), 6.87(d, J = 9.9 Hz, 1H, Ar-H), 3.91 (s, 3H, -OCH_3_), 3.90 (s, 3H, -OCH_3_), 3.66–3.68 (m, 1H, cyclopropyl), 3.33–3.55 (m, 16H, piperazinyl), 1.19–1.40 (m, 4H, cyclopropyl); ^13^C-NMR (CDCl_3_) δ 8.2, 35.3, 41.2, 41.2, 44.8, 44.8, 48.1, 49.5, 49.2, 50.3, 53.4, 54.2, 106.8, 107.9, 110.8, 112.4, 114.7, 119.1, 128.5, 128.1, 137.2, 146.3, 149.5, 153.4, 159.1, 160.6, 161.2, 165.8, 168.2, 176,4; FAB MS: m/z 608 [M+H]^+^; HRFABMS: calcd for C_31_H_35_FN_5_O_7_ [M+H]^+^ 608.2523, found 608.2525.

*1-Cyclopropyl-7-(4-[4-(2,4-dimethoxybenzoyl)piperazino]carbopiperazino)-6-fluoro-4-oxo-1,4-dihydro-3-quinolinecarboxylic acid* (**5h**). Amine **4** (0.67 g, 1.5 mmol) was treated with 2,4-dimethoxybenzoyl chloride (0.24 g, 1.2 mmol) to give **5h** (0.47 g, 65%) as a white solid; tlc R_f_ = 0.47 (DCM/EtOH = 20:1); mp 244–245 °C; UV λ_max_ (DCM/EtOH = 3 : 2) nm (log ε): 240.4 (9.29); ^1^H-NMR (CDCl_3_) δ 8.64 (s, 1H, C_2_-H), 7.88 (d, *J* = 12.9 Hz, 1H, C_5_-H), 7.33 (d, *J* = 7.2 Hz, 1H, C_8_-H), 7.17 (d, *J* = 8.4 Hz, 1H, Ar-H), 6.50 (dd, *J* = 8.4 Hz, 2.1 Hz, 1H, Ar-H), 6.43 (d, *J* = 2.1 Hz, 1H, Ar-H), 3.80 (s, 3H, -OCH_3_), 3.79 (s, 3H, -OCH_3_), 3.78–3.82 (m, 1H, cyclopropyl), 3.25–3.52 (m, 16H, piperaznyl), 1.18–1.39 (m, 4H, cyclopropyl); ^13^C-NMR (CDCl_3_) δ 8.2, 35.3, 41.2, 41.1, 44.6, 44.8, 47.3, 49.4, 49.2, 50.3, 53.4, 53.7, 106.8, 107.9, 110.8, 112.4, 114.7, 118.9, 127.2, 128.5, 137.8, 146.4, 148.2, 153.4, 159.3, 160.3, 161.2, 165.7, 168.1, 176,3; FABMS: m/z 608 [M+H]^+^; HRFABMS: calcd for C_31_H_35_FN_5_O_7_ [M+H]^+^ 608.2523, found 608.2519.

*1-Cyclopropyl-6-fluoro-7-(4-[4-(4-fluorobenzenesulfonyl)piperazino]carbopiperazino)-4-oxo-1,4-dihydro-3-quinolinecarboxylic acid* (**5i**). Amine **4** (0.67g, 1.5 mmol) was treated with 4-fluoro- benzenesulfonyl chloride (0.23 g, 1.2 mmol) to give **5i** (0.55 g, 76%) as a white solid; tlc R_f_ = 0.42 (DCM/EtOH = 20:1); mp 268–269 °C; UV λ_max_ (DCM/EtOH = 3:2) nm (log ε): 241.1 (9.24); ^1^H-NMR (CDCl_3_) δ 8.76 (s, 1H, C_2_-H), 8.02 (d, J = 12.9 Hz, 1H, C_5_-H), 7.77 (dt, J = 4.8 Hz, 2.1 Hz, 2H, Ar-H), 7.34 (d, J = 7.2 Hz, 1H, C_8_-H), 7.25 (dd, J = 4.8 Hz, 2.1 Hz, 2H, Ar-H), 3.02–3.51 (m, 17H, piperazinyl, cyclopropyl), 1.20–1.40 (m, 4H, cyclopropyl); ^13^C-NMR (CDCl_3_) δ 8.1, 35.3, 41.4, 41.9, 44.3, 44.5, 47.9, 49.3, 49.6, 50.4, 107.1, 108.7, 111.3, 112.2, 112.6, 114.4, 127.3, 128.2, 137.8, 138.6, 146.5, 149.8, 153.4, 158.2, 161.9, 165.8, 176,5; FABMS: m/z 602 [M+H]^+^; HRFABMS: calcd for C_28_H_30_F_2_N_5_O_6_S [M+H]^+^ 602.1887, found 602.1885.

*7-(4-[4-(4-Chlorobenzenesulfonyl)piperazino]carbopiperazino)-1-cyclopropyl-6-fluoro-4-oxo-1,4-dihydro-3-quinolinecarboxylic acid* (**5j**). Amine **4** (0.67 g, 1.5 mmol) was treated with 4-chlorobenzenesulfonyl chloride (0.25 g, 1.2 mmol) to give **5j** (0.58 g, 78%) as a white solid; tlc R_f_ = 0.42 (DCM/EtOH = 20:1); mp 274–275 °C; UV λ_max_ (DCM/EtOH = 3:2) nm (log ε): 242.5 (9.29); ^1^H-NMR (CDCl_3_) δ 8.74 (s, 1H, C_2_-H), 8.00 (d, J = 12.9 Hz, 1H, C_5_-H), 7.69 (d, J = 8.4 Hz, 2H, Ar-H), 7.53 (d, J = 8.4 Hz, 2H, Ar-H), 7.33 (d, J = 7.2 Hz, 1H, C_8_-H), 3.04–3.47 (m, 17H, piperazinyl, cyclopropyl), 1.20–1.40 (m, 4H, cyclopropyl); ^13^C-NMR (CDCl_3_) δ 8.1, 35.3, 41.4, 41.9, 44.3, 44.5, 47.9, 49.3, 49.6, 50.4, 107.1, 108.7, 111.3, 112.6, 127.3, 128.2, 128.9, 129.3, 135.6, 137.8, 138.6, 146.5, 149.8, 153.4, 161.9, 165.8, 176,4; FABMS: m/z 618 [M+H]^+^; HRFABMS: calcd for C_28_H_30_FClN_5_O_6_S [M+H]^+^ 618.1592, found 618.1589.

*1-Cyclopropyl-6-fluoro-4-oxo-7-(4-[4-(4-trifluoromethoxybenzenesulfonyl)piperazino]carbopiperazino)-1,4-dihydro-3-quinolinecarboxylic acid* (**5k**). Amine **4** (0.67 g, 1.5 mmol) was treated with 4-trifluoromethoxybenzenesulfonyl chloride (0.31 g, 1.2 mmol) to give **5k** (0.64 g, 80%) as a white solid; tlc R_f_ = 0.40 (DCM/EtOH = 20:1); mp 248–249 °C; UV λ_max_ (DCM/EtOH = 3 : 2) nm (log ε): 239.2 (9.33); ^1^H-NMR (CDCl_3_) δ 8.77 (s, 1H, C_2_-H), 8.03 (d, J = 12.9 Hz, 1H, C_5_-H), 7.81 (d, J = 8.7 Hz, 2H, Ar-H), 7.38 (d, J = 8.7 Hz, 2H, Ar-H), 7.35 (d, J = 7.2 Hz, 1H, C_8_-H), 3.06–3.46 (m, 17H, piperazinyl, cyclopropyl), 1.19–1.40 (m, 4H, cyclopropyl); ^13^C-NMR (CDCl_3_) δ 8.2, 35.2, 41.6, 41.9, 44.2, 44.5, 47.9, 49.8, 49.5, 50.4, 107.2, 108.7, 111.3, 112.6, 114.7, 119.3, 127.3, 128.2, 137.8, 138.6, 146.5, 149.2, 151.8, 158.2, 160.4, 161.5, 165.4, 176,2; FABMS: m/z 668 [M+H]^+^; HRFABMS: calcd for C_29_H_30_F_4_N_5_O_7_S [M+H]^+^ 668.1804, found 668.1802.

*1-Cyclopropyl-6-fluoro-4-oxo-7-(4-[4-(4-trifluoromethylbenzenesulfonyl)piperazino]carbopiperazino)-1,4-dihydro-3-quinolinecarboxylic acid* (**5l**). Amine **4** (0.67 g, 1.5 mmol) was treated with 4-trifluoromethylbenzenesulfonyl chloride (0.29 g, 1.2 mmol) to give **5l** (0.61 g, 78%); tlc R_f_ = 0.40 (DCM/EtOH = 20:1); mp 167–168 °C; UV λ_max_ (DCM/EtOH = 3:2) nm (log ε): 241.1 (9.28); ^1^H-NMR (DMSO-*d_6_* with a drop of D_2_O) δ 8.64 (s, 1H, C_2_-H), 8.04 (d, J = 8.4 Hz, 2H, Ar-H), 7.96 (d, J = 8.4 Hz, 2H, Ar-H), 7.87 (d, J = 13.2 Hz, 1H, C_5_-H), 7.51 (d, J = 7.5 Hz, 1H, C_8_-H), 3.74–3.80 (m, 1H, cyclopropyl), 2.96–3.30 (m, 16H, piperazinyl), 1.12–1.30 (m, 4H, cyclopropyl); ^13^C-NMR (CDCl_3_) δ 8.2, 35.1, 41.6, 41.9, 44.3, 44.5, 47.9, 49.3, 49.6, 50.4, 107.3, 108.7, 111.3, 112.2, 124.5, 125.6, 126.2, 127.3, 128.2, 130.8, 137.8, 138.6, 145.7, 149.2, 158.7, 161.3, 165.4, 176,2; FABMS: m/z 652 [M+H]^+^; HRFABMS: calcd for C_29_H_30_F_4_N_5_O_6_S [M+H]^+^ 652.1855, found 652.1853.

*1-Cyclopropyl-6-fluoro-4-oxo-7-(4-[4-(3-trifluoromethylbenzenesulfonyl)piperazino]carbopiperazino)-1,4-dihydro-3-quinolinecarboxylic acid* (**5m**). Amine **4** (0.67 g, 1.5 mmol) was treated with 3-trifluoromethylbenzenesulfonyl chloride (0.29 g, 1.2 mmol) to give **5m** (0.59 g, 76%) as a white solid; tlc R_f_ = 0.37 (DCM/EtOH = 20:1); mp 267–268 °C; UV λ_max_ (DCM/EtOH = 3:2) nm (log ε): 239.2 (9.32); ^1^H-NMR (DMSO-*d_6_* with a drop of D_2_O) δ 8.64 (s, 1H, C_2_-H), 8.15 (d, J = 7.5 Hz, 1H, Ar-H), δ 8.07 (d, J = 7.8 Hz, 1H, Ar-H), δ 7.97 (s, 1H, Ar-H), δ 7.92 (dd, J = 7.8 Hz, 7.5 Hz, 1H, Ar-H), 7.89 (d, J = 13.2 Hz, 1H, C_5_-H), 7.52 (d, J = 7.5 Hz, 1H, C_8_-H), 3.76 ~ δ 3.81 (m, 1H, cyclopropyl), 2.97–3.30 (m, 16H, piperazinyl), 1.13–1.30 (m, 4H, cyclopropyl); ^13^C-NMR (CDCl_3_) δ 8.1, 35.4, 41.2, 41.9, 44.4, 44.9, 47.5, 49.3, 49.6, 50.4, 107.3, 108.1, 111.5, 112.2, 124.1, 125.2, 126.6, 127.4, 128.1, 130.2, 137.8, 138.6, 145.1, 149.6, 158.7, 161.6, 165.2, 176,2; FABMS: m/z 652 [M+H]^+^; HRFABMS: calcd for C_29_H_30_F_4_N_5_O_6_S [M+H]^+^ 652.1855, found 652.1852.

### 3.3. Antimicrobial Susceptibility Testing

*Escherichia coli* BCRC 13084 and *Pseudomonas aeruginosa* ATCC 27853 were obtained from the Culture Collection and Research Center (CCRC), Hsin-Chu, Taiwan, R.O.C. Clinical isolates of *Bacillus subtilis*, methicillin-resistant *Staphylococcus aureus* (MRSA) and *ciprofloxacin-resistant* (*cipro*^r^) *Pseudomonas aeruginosa* (CRPA) were obtained from the Culture Collection of Tri-Service General Hospital, Taipei, Taiwan. *Escherichia coli* JM109 harboring plasmid pUC 18 (*E. coli*/pUC18) was kindly provided by Department of Microbiology and Immunology, National Defense Medical Center, Taiwan and used as a *beta*-lactamase-producing clone. All bacteria were stored in Luria-Bertani broth (Difco Laboratories, Detroit, MI, USA) with 15% glycerol (vol/vol) at −80 °C.

To determine the efficacies of minimum inhibitory and bactericidal concentrations (MICs and MBCs) of the synthetic derivatives of ciprofloxacin, a wide range of Gram-negative (*E. coli*, *E. coli*/pUC18, and *Pseudomonas aeruginosa*) and Gram-positive (MRSA and *Bacillus subtilis*) bacterial species were used by the broth microdilution technique as described by the Clinical and Laboratory Standards Institute (CLSI) methodology [14]. In addition to the synthesized compounds, the prevailing antibiotics, including norfloxacin and ciprofloxacin (Sigma Chemical Co., St. Louis, MO, USA) were included for comparison. Serial two-fold dilutions, ranging from 0.016 to 256 μg/mL, for each antibiotic in Mueller-Hinton broth (Difco) were prepared in 96-well flat-bottom polystyrene microtiter plates. Each compound for each organism with an inoculum size of 2 × 10^5^ CFUs was carried out and cultures were incubated at 37 °C for 24 h. The MICs were recorded as the lowest concentration that produced inhibition of visible growth after overnight incubation. Each experiment was performed independently three times. MBCs were obtained by sampling ten-microliter cultures from each well and streaking onto the surface of Mueller-Hinton agar plates. After overnight incubation at 37 °C, colonies were counted and the MBCs, defined as the concentration at which did not show any bacterial growth after incubation during MIC assay, were determined [15]. Each experiment was also performed independently three times.

## 4. Conclusions

Modifications to the structure of ciprofloxacin at the 7-position has provided 7-(4-carbo- piperazin-4-yl) derivatives with improved antimicrobial activities compared to the prevailing ciprofloxacin. 7-[4-Carbopiperazin-4-(3,5-dimethoxybenzoyl)-yl] derivative **5h** and 7-[4-carbo- piperazin-4-(4-trifluoromethoxybenzenesulfonyl)-yl] derivative **5k** showed an impressive selective potency against a clinic isolate of CRPA and also maintained activities against MRSA, demonstrating that the delicate manipulation at the 7-position of the fluoroquinolone framework can still be a suitable ways to obtain new, broad spectrum fluoroquinolones, against especially the Gram-negative pathogens and certain drug-resistant strains.

## Supplementary Materials

Supplementary File 1
