# Supplementary material for: New 7-[4-(4-(un)Substituted)piperazine-1-carbonyl]-piperazin-1-yl] Derivatives of Fluoroquinolone: Synthesis and Antimicrobial Evaluation
Source: Molecules. 2013 Jun 27;18(7):7557–69. doi: 10.3390/molecules18077557 (PMC6269787; doi:10.3390/molecules18077557)
Supplement: Supplementary File 1 [file molecules-18-07557-s001.pdf]

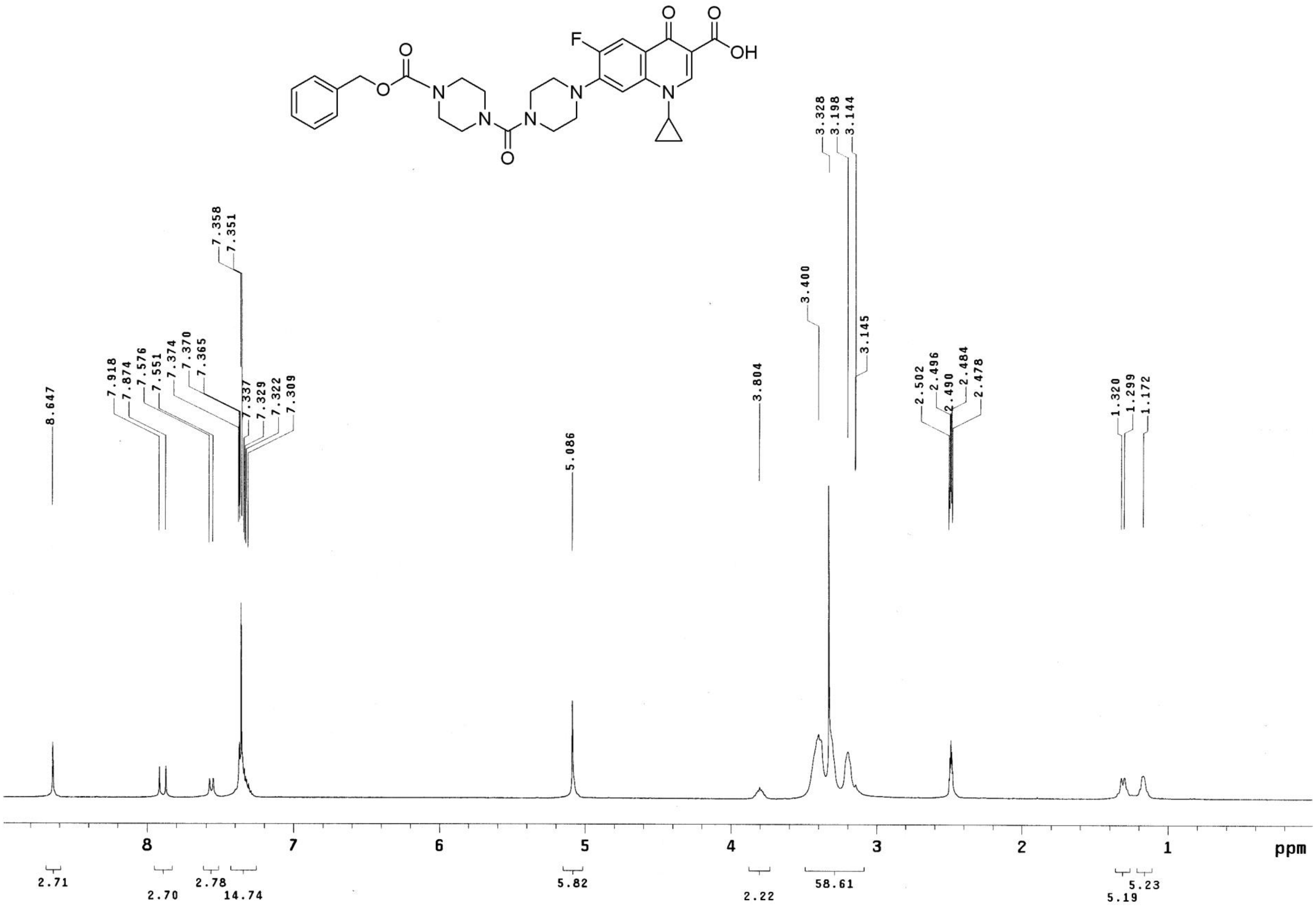

^{1}H-NMR (300 MHz, DMSO-*d6*) spectrum of **3**

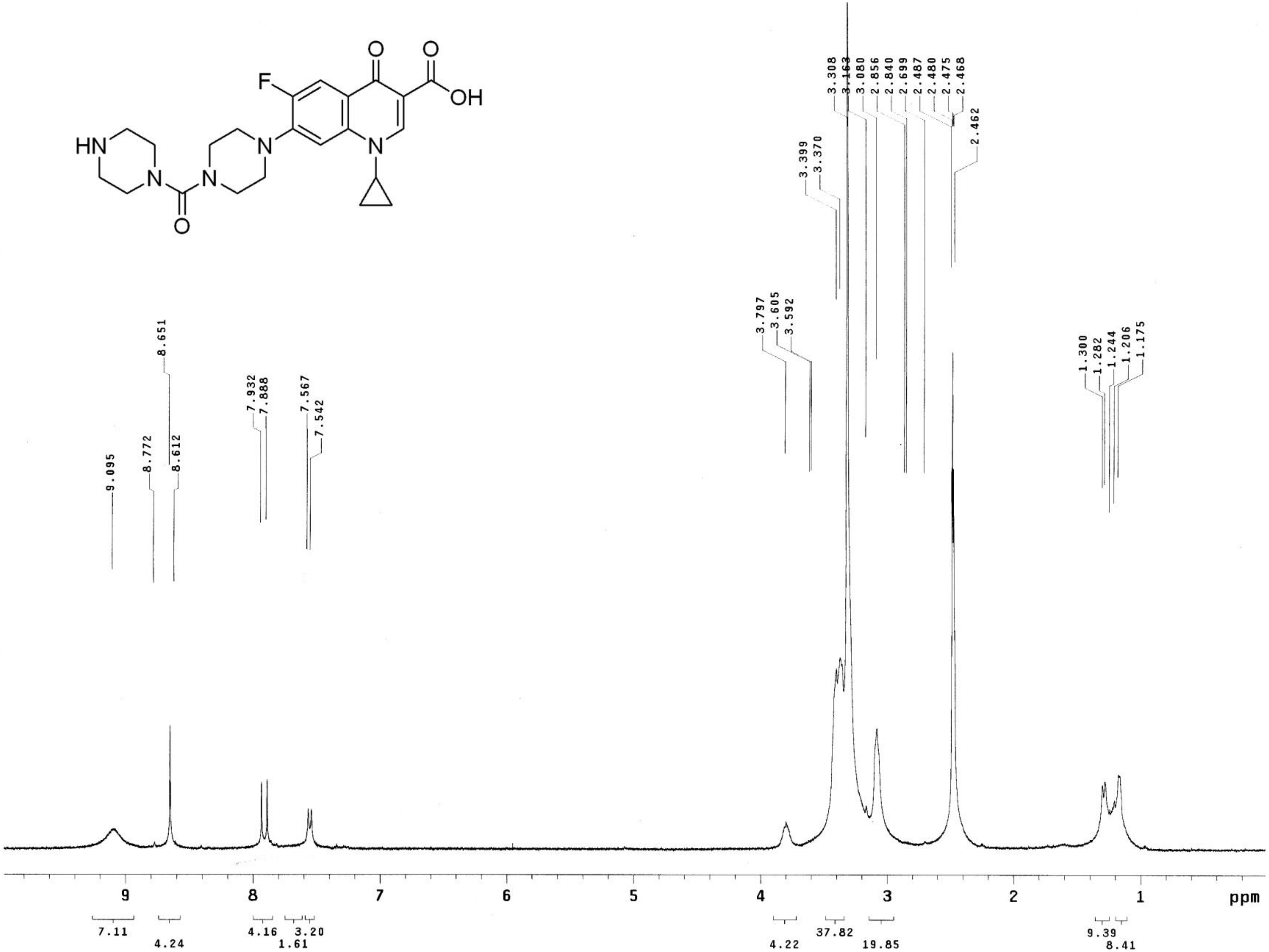

^{1}H-NMR (300 MHz, DMSO-*d6*) spectrum of**4**

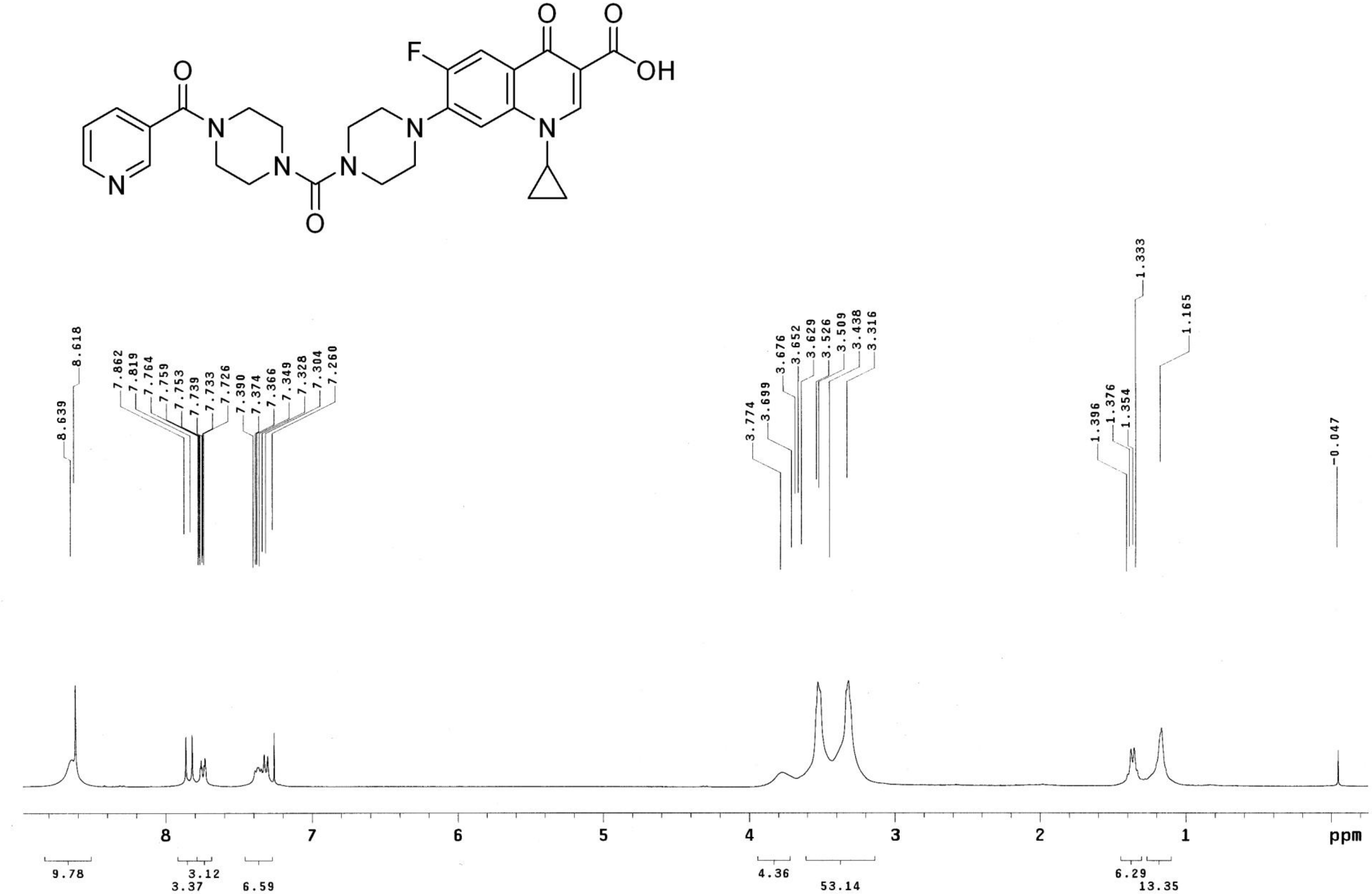

^{1}H-NMR (300 MHz, $CDCl_3$) spectrum of **5a**

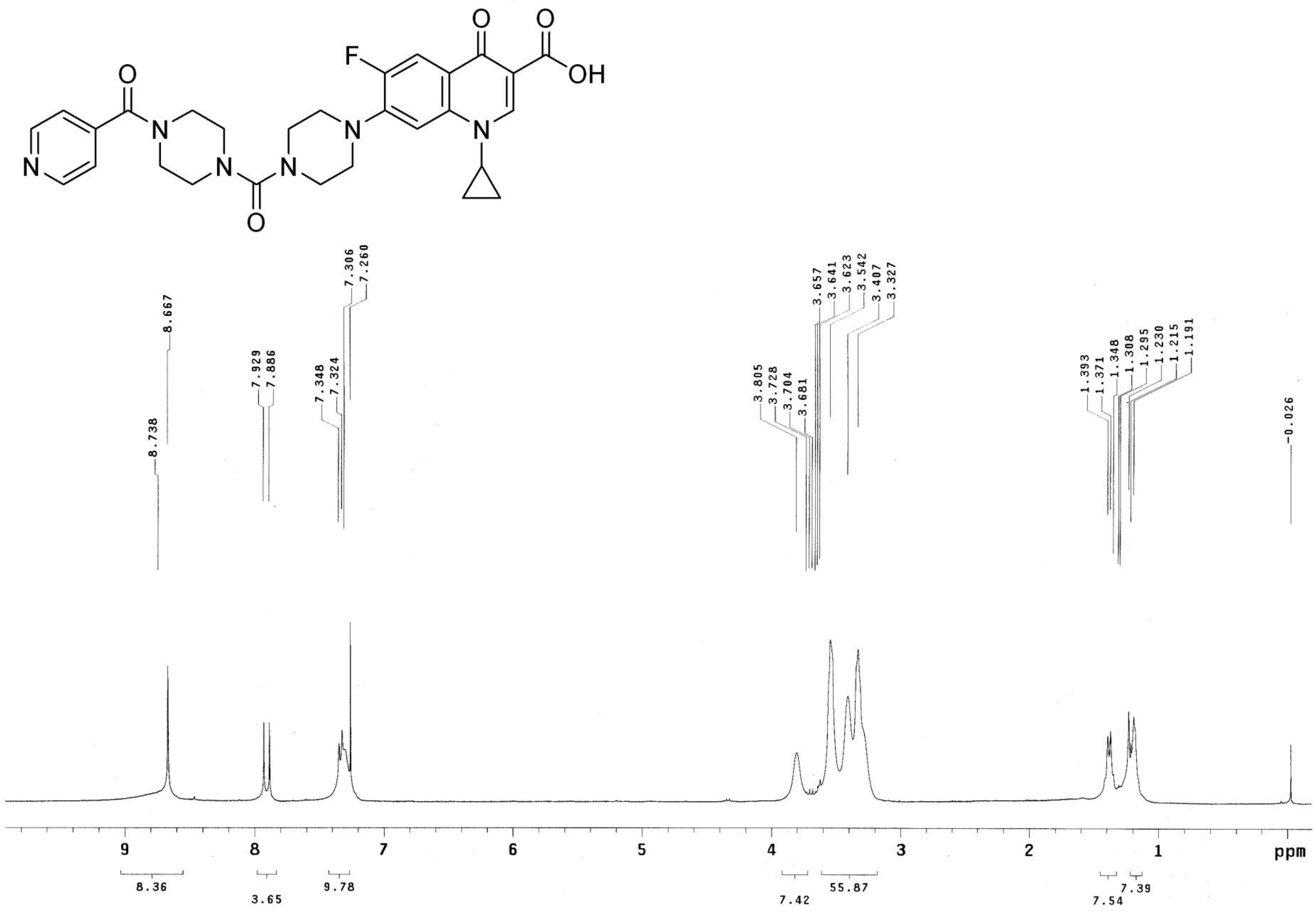

^{1}H-NMR (300 MHz, $CDCl_3$) spectrum of **5b**

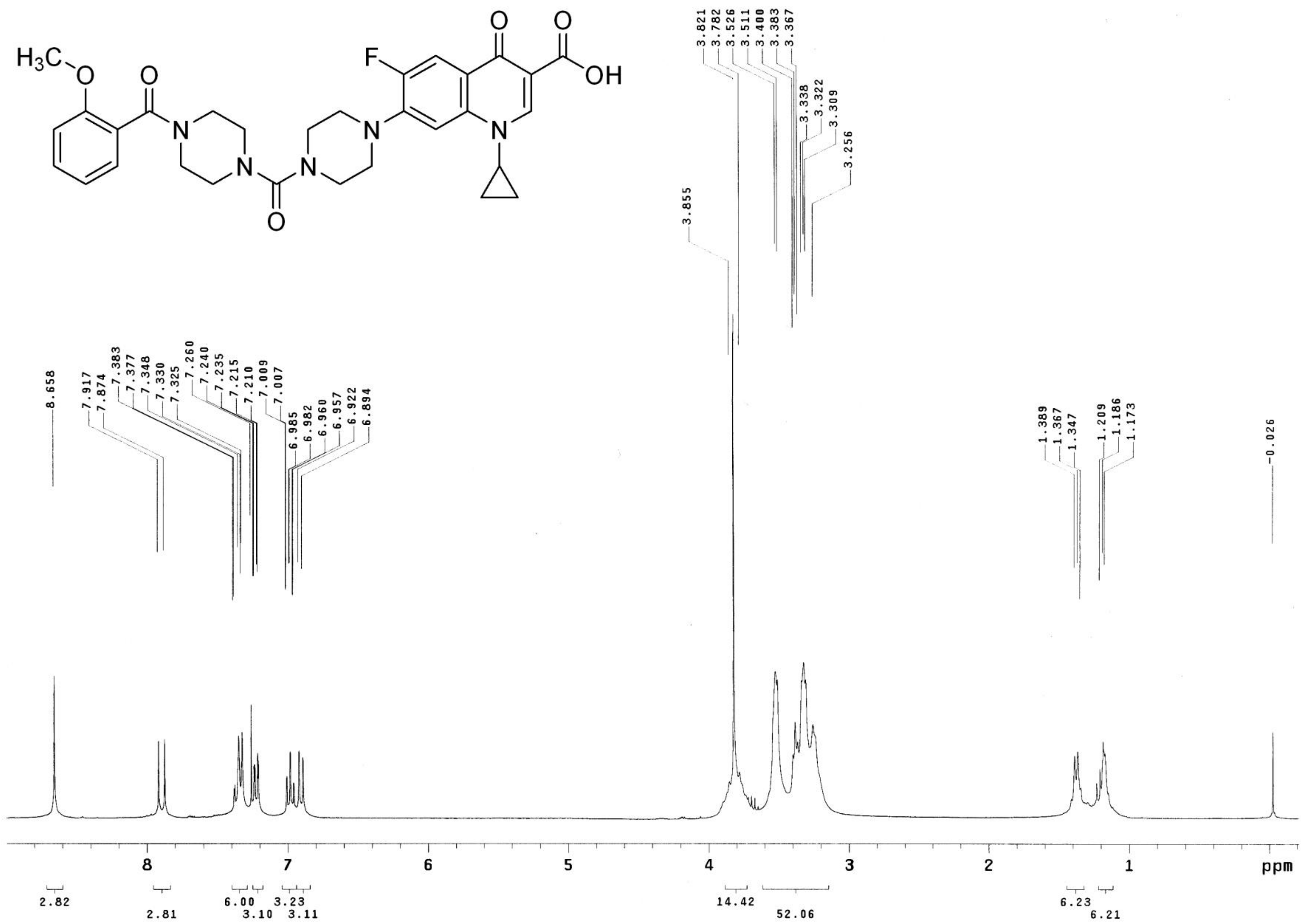

^{1}H-NMR (300 MHz, $CDCl_3$) spectrum of **5c**

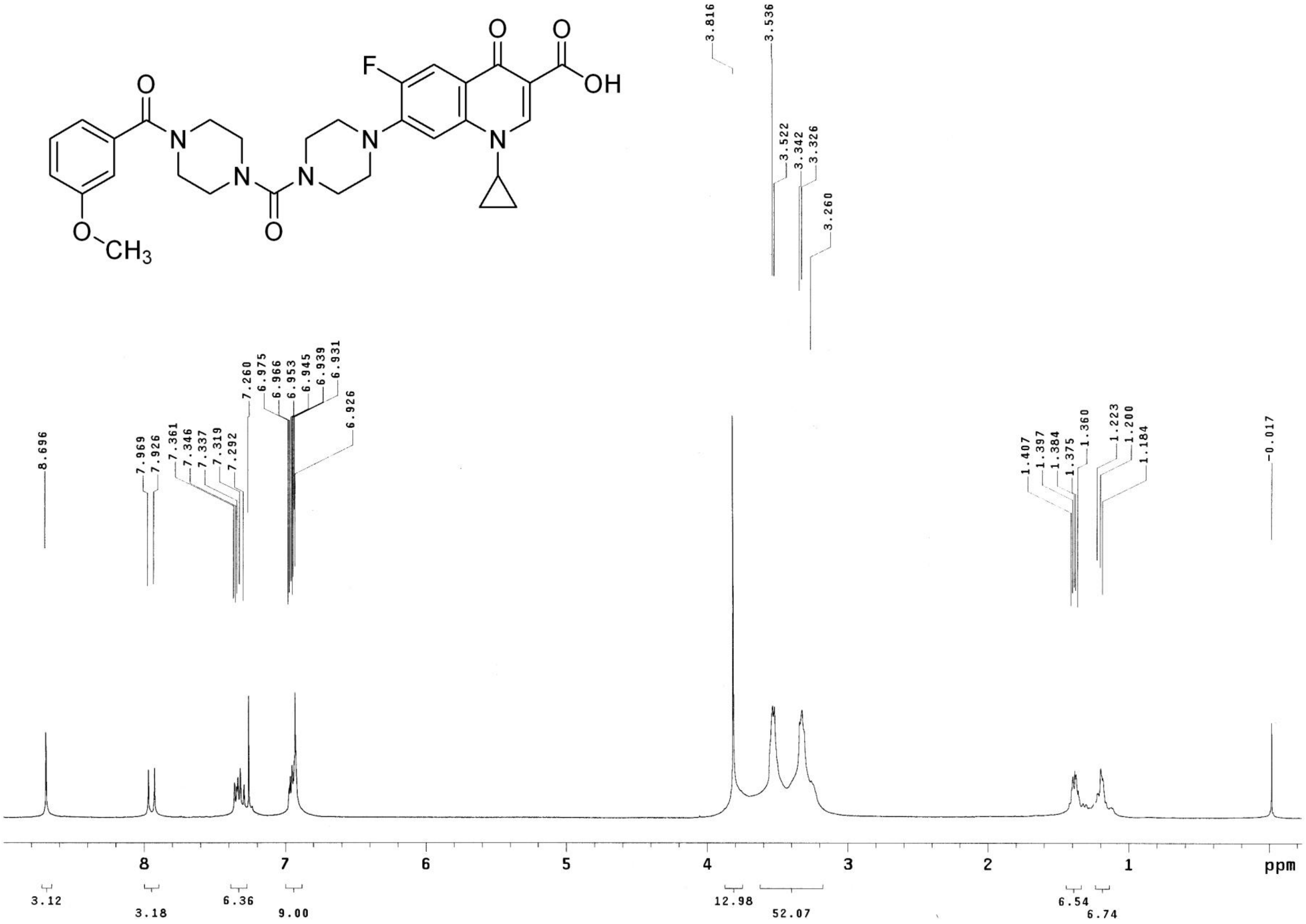

^{1}H-NMR (300 MHz, $CDCl_3$) spectrum of **5d**

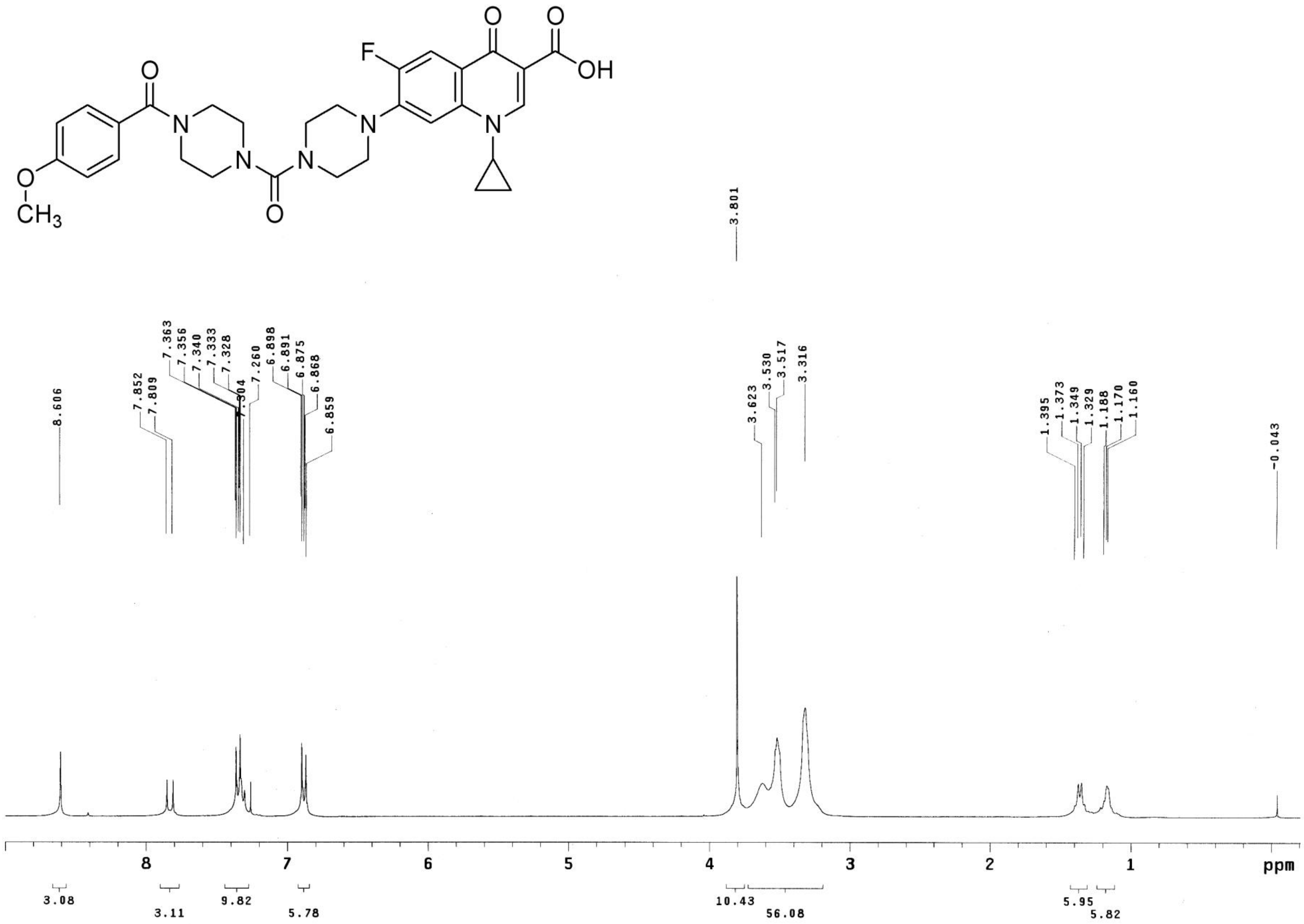

^{1}H-NMR (300 MHz, $CDCl_3$) spectrum of **5e**

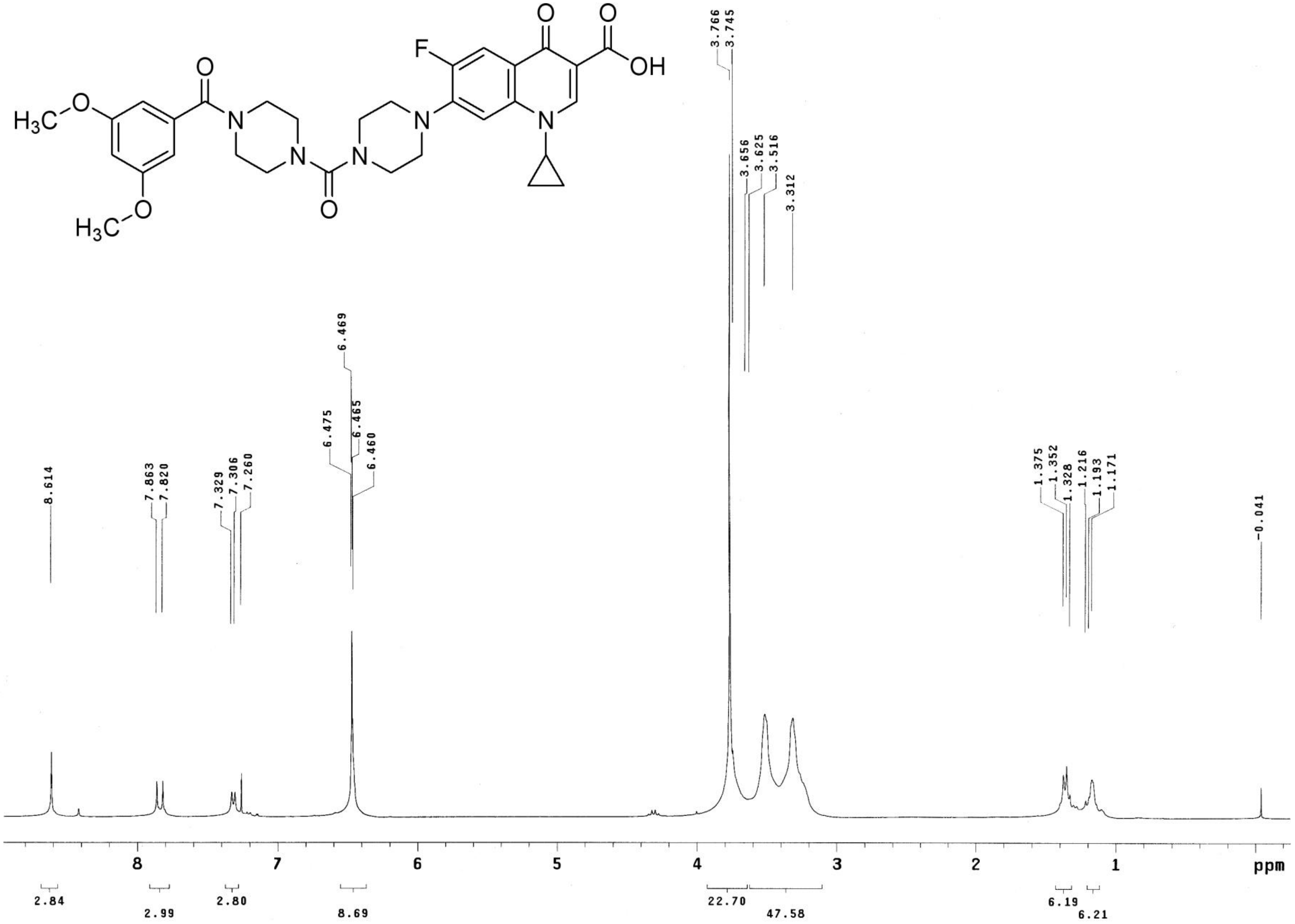

^{1}H-NMR (300 MHz, $CDCl_3$) spectrum of **5f**

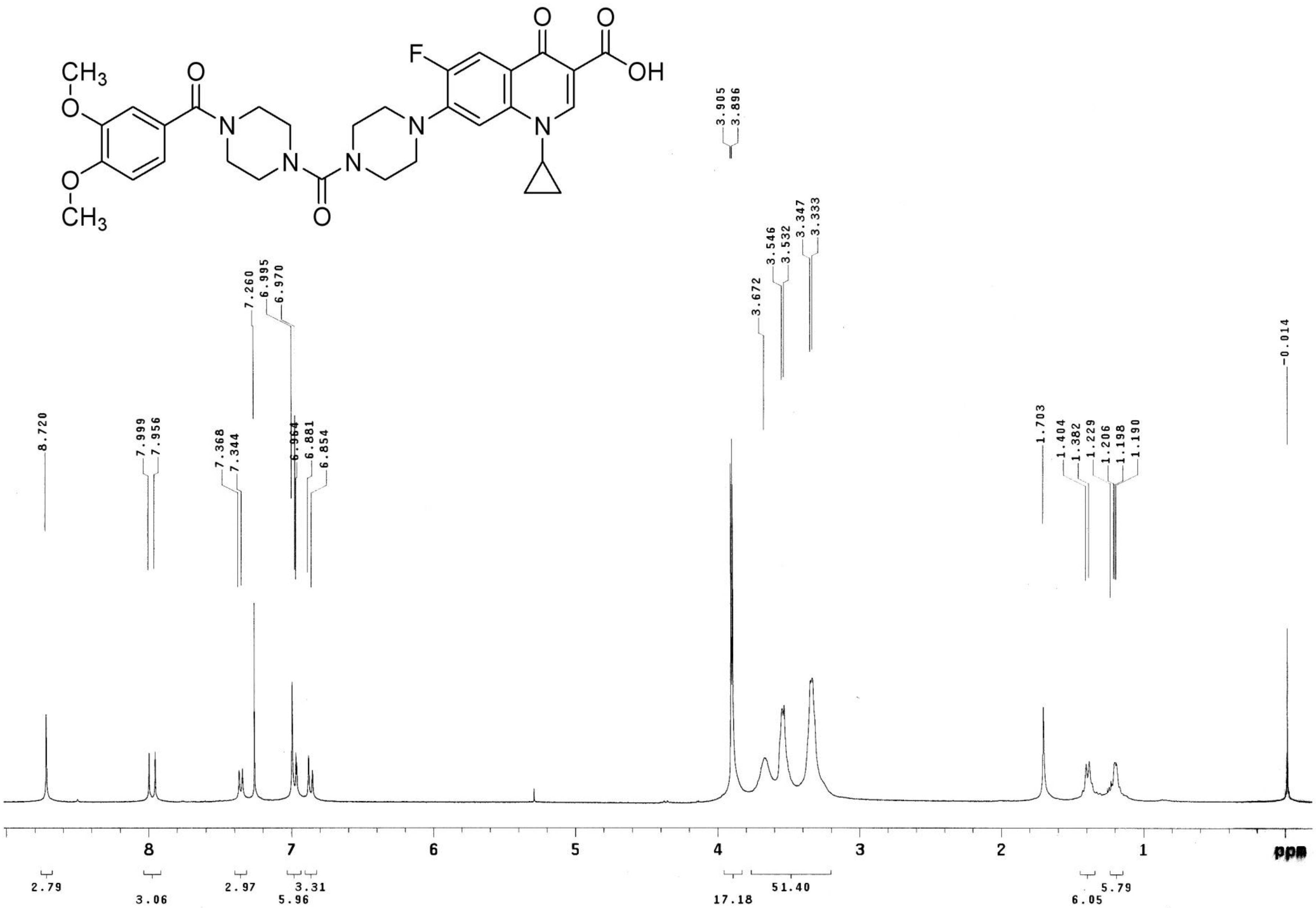

^{1}H-NMR (300 MHz, $CDCl_3$) spectrum of **5g**

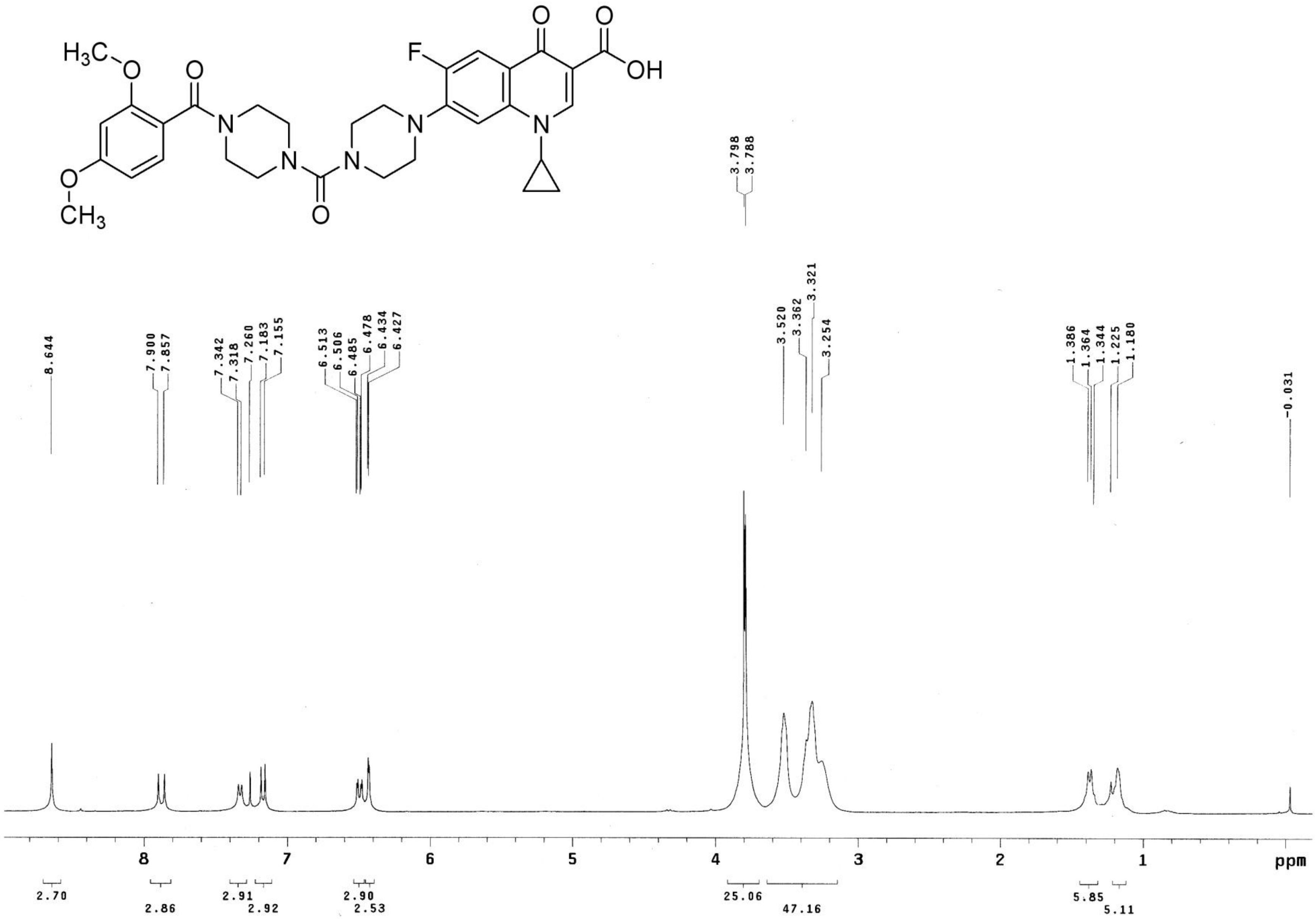

^{1}H-NMR (300 MHz, $CDCl_3$) spectrum of **5h**

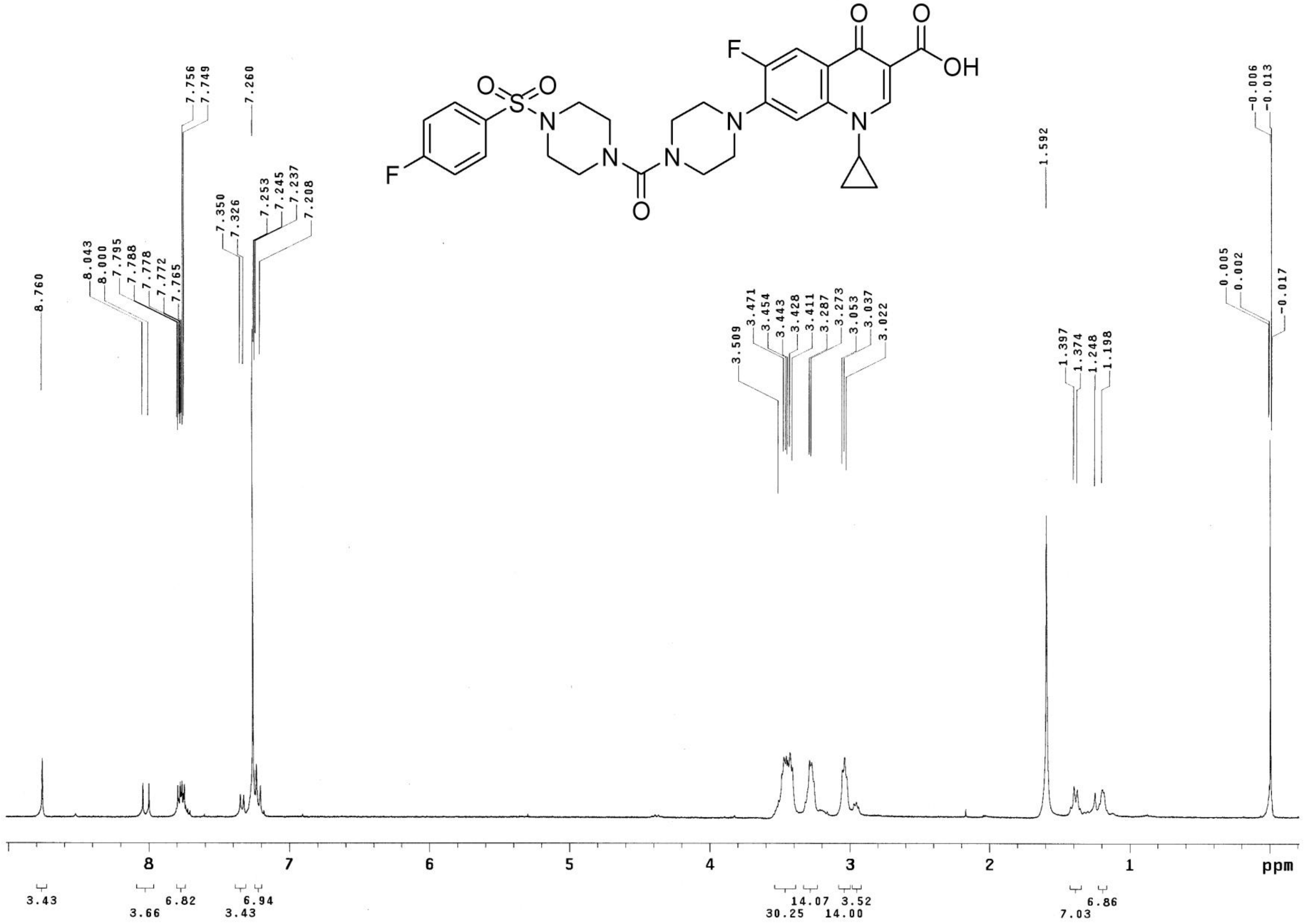

^{1}H-NMR (300 MHz, $CDCl_3$) spectrum of **5i**

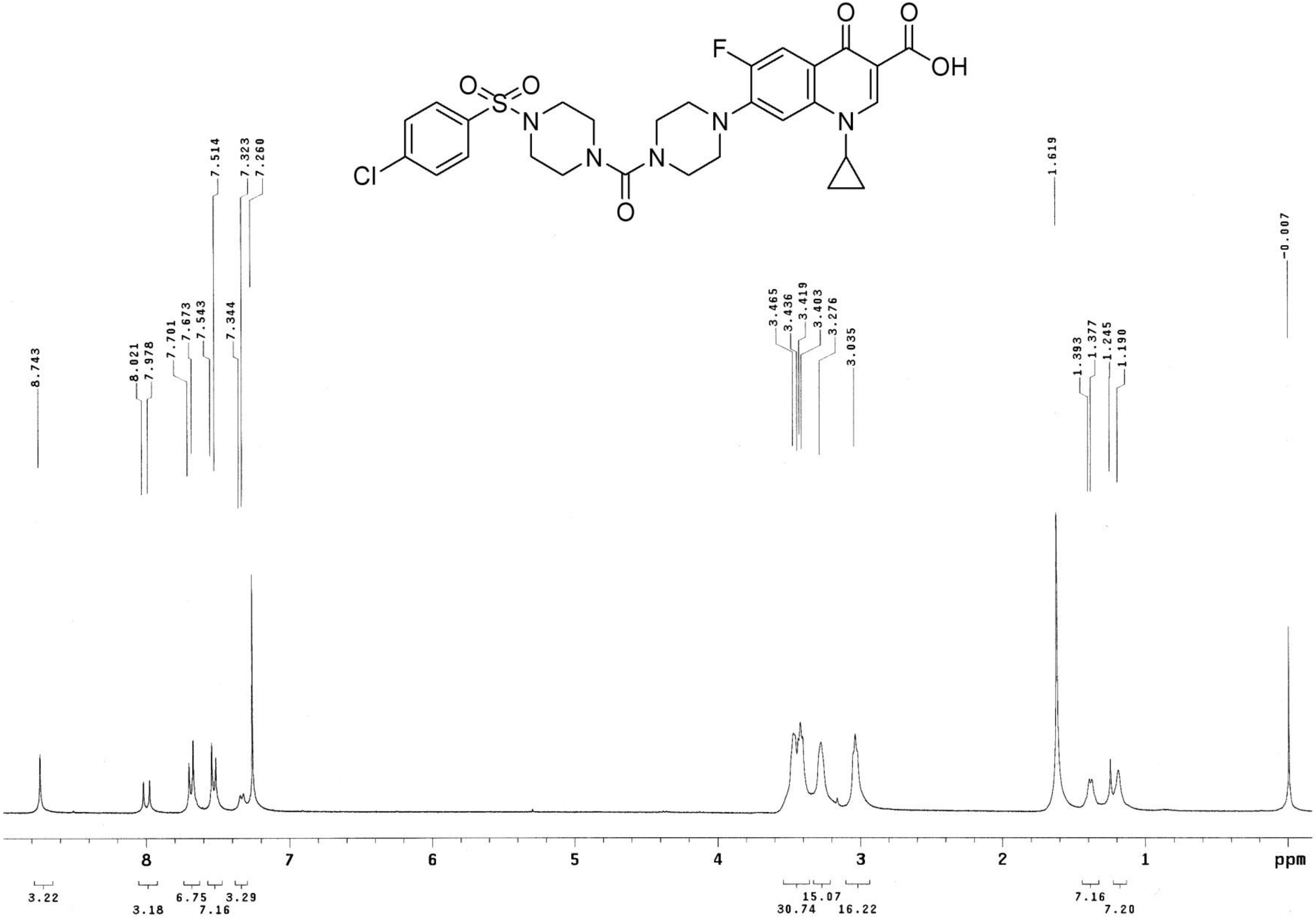

^{1}H-NMR (300 MHz, $CDCl_3$) spectrum of **5j**

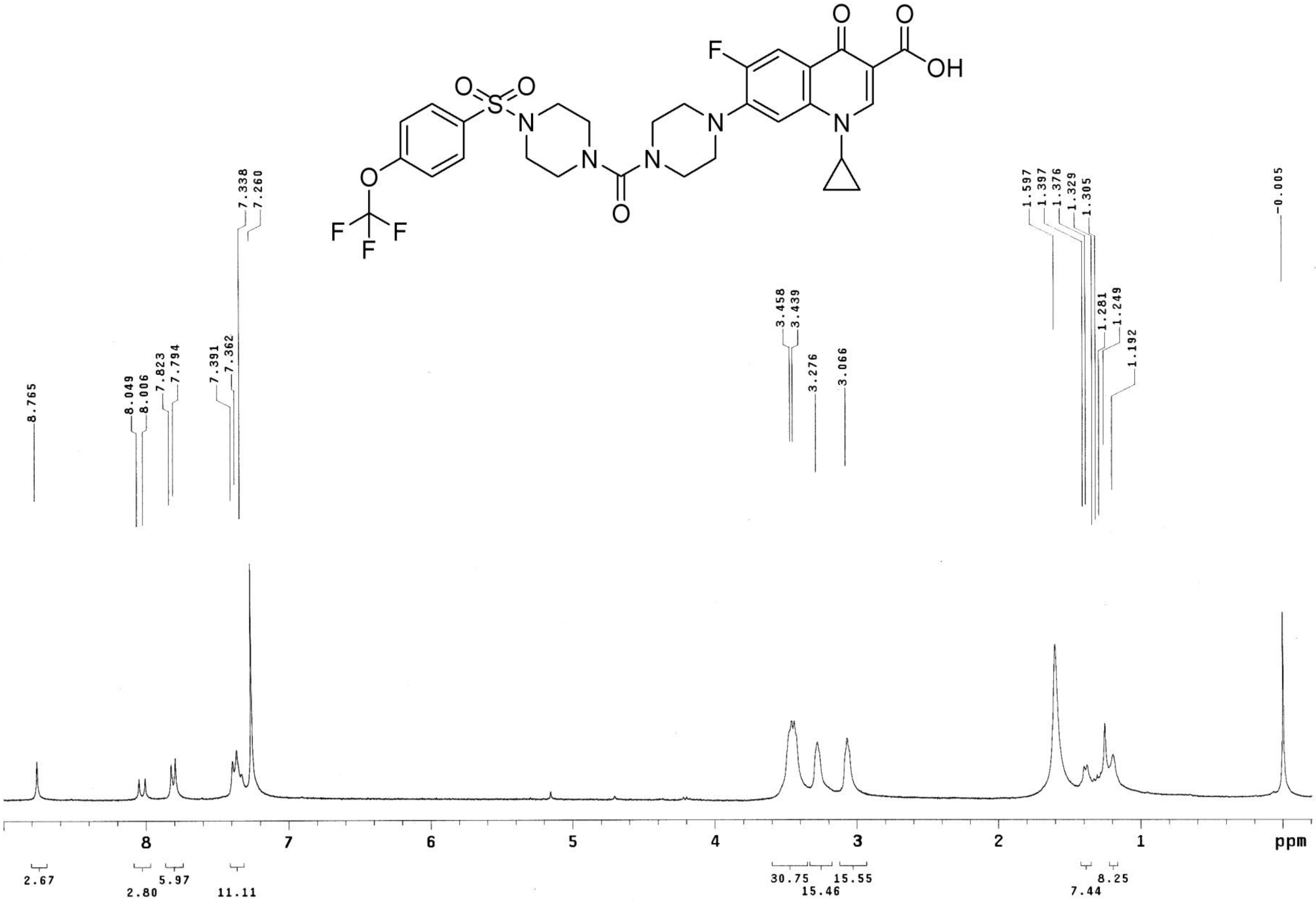

^{1}H-NMR (300 MHz, $CDCl_3$) spectrum of **5k**

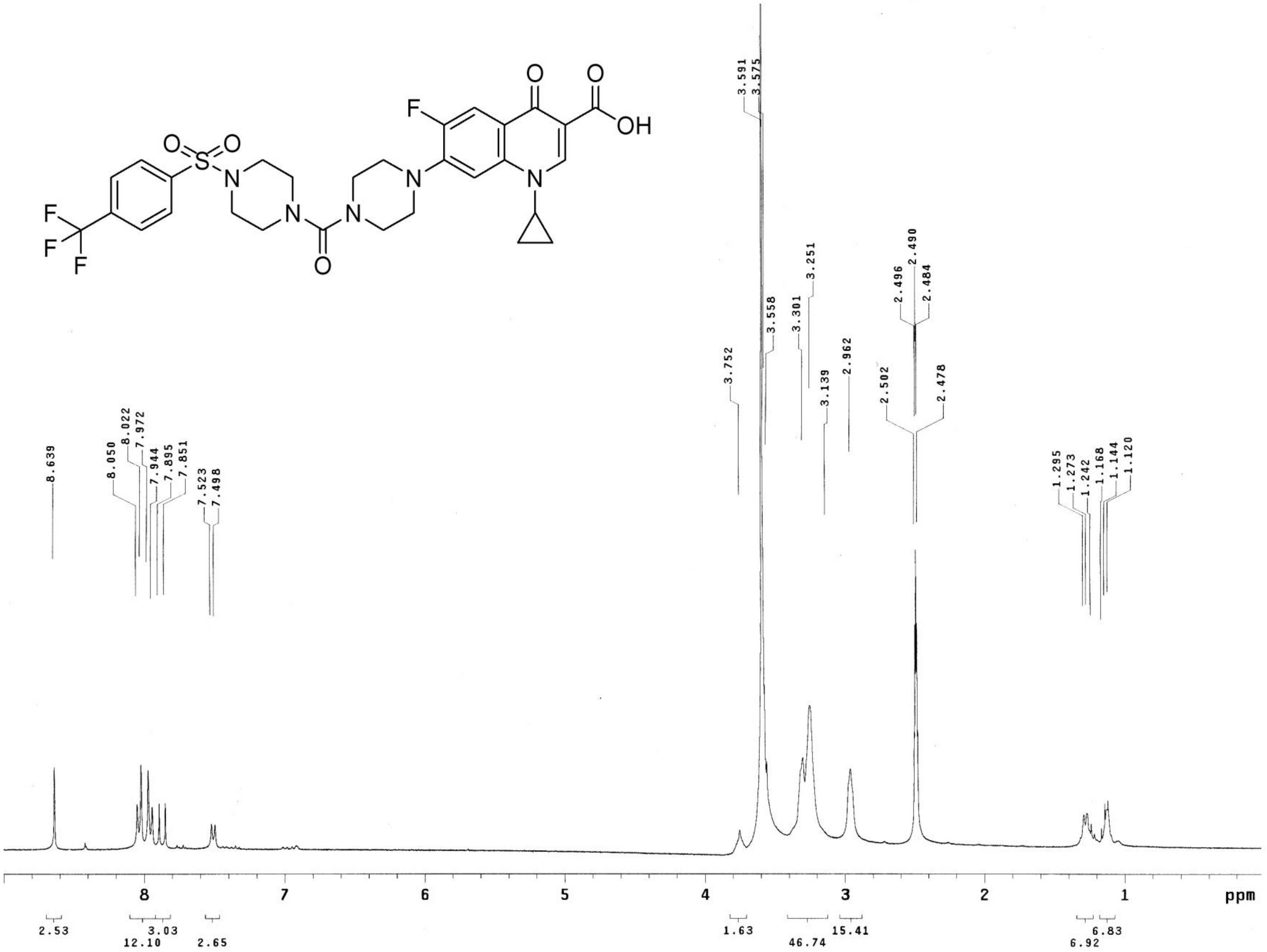

^{1}H-NMR (300 MHz, DMSO-*d6* with a drop of D_2O) spectrum of **5l**

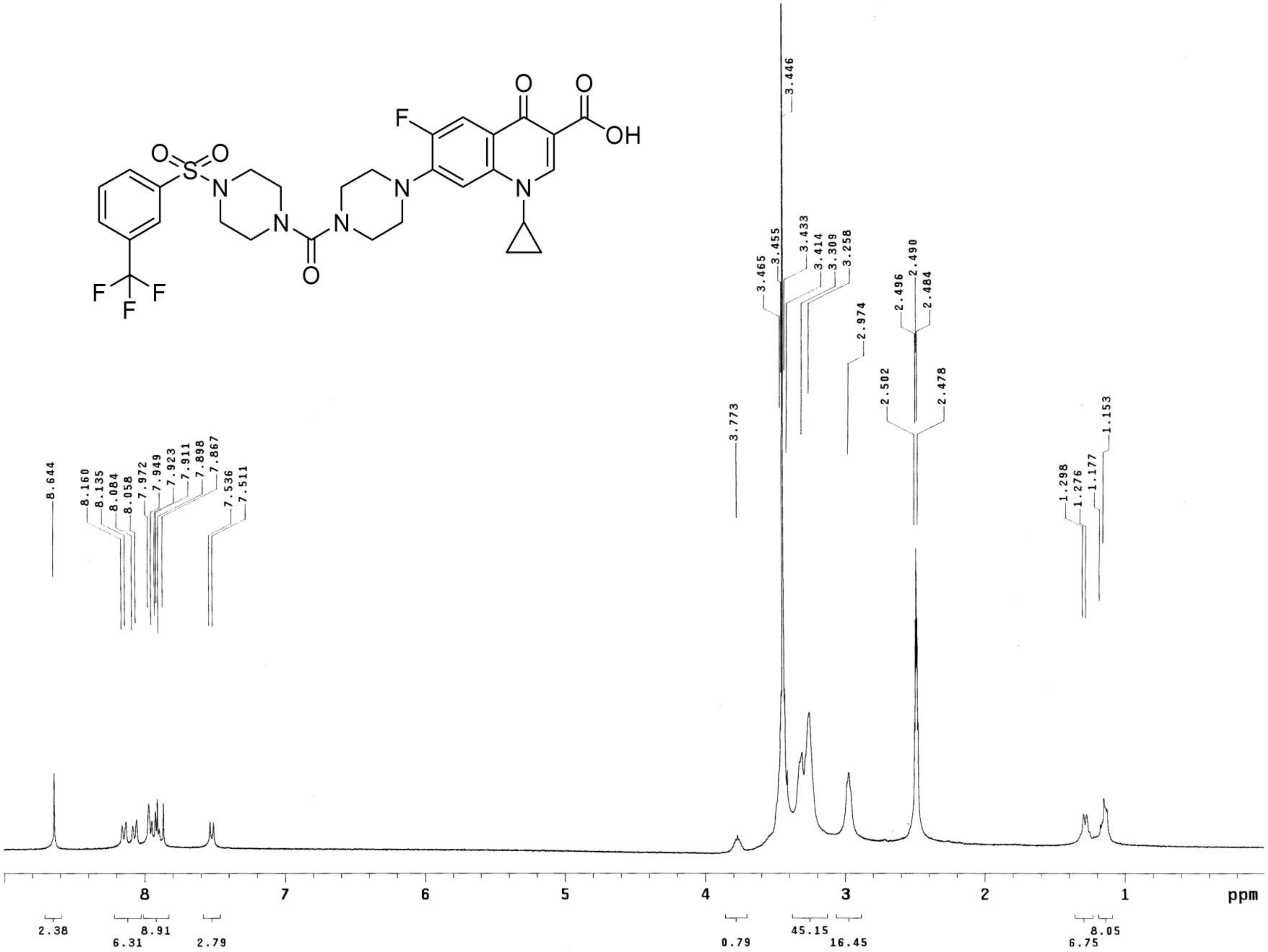

^{1}H-NMR (300 MHz, DMSO-*d6* with a drop of D_2O) spectrum of **5m**
